# A flexible and parallelizable approach to genome‐wide polygenic risk scores

**DOI:** 10.1002/gepi.22245

**Published:** 2019-07-22

**Authors:** Paul J. Newcombe, Christopher P. Nelson, Nilesh J. Samani, Frank Dudbridge

**Affiliations:** ^1^ MRC Biostatistics Unit, School of Clinical Medicine, Cambridge Institute of Public Health Cambridge Biomedical Campus Cambridge UK; ^2^ Department of Cardiovascular Sciences, Cardiovascular Research Centre, Glenfield Hospital University of Leicester Leicester UK; ^3^ NIHR Leicester Biomedical Research Centre Glenfield Hospital Leicester UK; ^4^ Department of Health Sciences, Centre for Medicine University of Leicester Leicester UK

**Keywords:** Bayesian variable selection, meta‐GWAS, polygenic risk scores, risk prediction, summary statistics

## Abstract

The heritability of most complex traits is driven by variants throughout the genome. Consequently, polygenic risk scores, which combine information on multiple variants genome‐wide, have demonstrated improved accuracy in genetic risk prediction. We present a new two‐step approach to constructing genome‐wide polygenic risk scores from meta‐GWAS summary statistics. Local linkage disequilibrium (LD) is adjusted for in Step 1, followed by, uniquely, long‐range LD in Step 2. Our algorithm is highly parallelizable since block‐wise analyses in Step 1 can be distributed across a high‐performance computing cluster, and flexible, since sparsity and heritability are estimated within each block. Inference is obtained through a formal Bayesian variable selection framework, meaning final risk predictions are averaged over competing models.

We compared our method to two alternative approaches: LDPred and lassosum using all seven traits in the Welcome Trust Case Control Consortium as well as meta‐GWAS summaries for type 1 diabetes (T1D), coronary artery disease, and schizophrenia. Performance was generally similar across methods, although our framework provided more accurate predictions for T1D, for which there are multiple heterogeneous signals in regions of both short‐ and long‐range LD. With sufficient compute resources, our method also allows the fastest runtimes.

## 1 INTRODUCTION

The heritability of most complex traits is driven by variation throughout the genome, with a large number of loci contributing small or modest effects (Dudbridge, 2013; 2016). Polygenic risk scores, which combine information on multiple variants genome‐wide into weighted sums of trait‐associated alleles, have been found to improve prediction of a variety of traits (Dudbridge, 2013; Evans, Visscher, & Wray, 2009; Pharoah, Antoniou, Easton, & Ponder, 2008; Purcell et al., 2009; Stahl et al., 2012; The International Multiple Sclerosis Genetics Consortium (IMSGC), 2010). Even for a modestly heritable trait such as breast cancer, a comprehensive polygenic score could improve discriminatory power sufficiently for use in a targeted screening program (Pharoah et al., 2002). However, the predictive accuracy of current GWAS “hits” falls well short of what is theoretically possible based on familial and genomic heritability estimates (De Vries et al., 2015; Eriksson et al., 2015; Talmud et al., 2015; Wacholder et al., 2010). Realizing the full potential of polygenic risk prediction will require much larger sample sizes than offered by a typical single cohort GWAS (Chatterjee et al., 2013; Dudbridge, 2013; Wray et al., 2013). In a recent trend large‐scale “meta‐GWAS”, comprising 10s of 1000s of people amassed over multiple studies, have boosted the power of genetic association studies, increasing the number of unambiguously associated regions into the 10s or even 100s for some traits (Lango Allen et al., 2010; Morris et al., 2012; Teslovich et al., 2010). Building predictive models from meta‐GWAS results is therefore of great importance, since these consortia often represent the totality of GWAS information for a particular trait.

A simple yet surprisingly effective approach is to select a subset of variants according to a *p*‐value threshold and build an additive score, weighting the contribution of individual variants according to their associations with the trait. This is easy to do from summary results alone, and requires minimal computation. The main drawback is that genetic correlations due to linkage disequilibrium (LD) are ignored, which leads to bias in the weights, and, consequently, suboptimal predictive accuracy. Ideally, multivariate regression would be used to estimate weights adjusted for LD. Unfortunately, however, this is complicated for two reasons. The first is that privacy concerns and the logistics of sharing data on such a large scale mean that meta‐GWAS typically only conduct one‐at‐a‐time association tests of each variant, based on simple summaries shared between the cohorts. The final univariate results ignore correlations among the variants, and consequently signals tagged by multiple correlated variants would be overrepresented in a polygenic risk score constructed according to a simple threshold on statistical significance. This issue is usually dealt with by pruning variants until they are approximately independent, although information is inevitably discarded (Dudbridge & Newcombe, 2015). The second issue relates to the challenge of dimensionality. Ideally, we would jointly model all important predictors, in order to account for genetic correlations due to LD. However, traditional regression methodology suffers from over‐fitting when applied to large numbers of covariates; information is spread too thinly leading to unstable estimates with high standard errors. This latter issue inspired the development of lasso penalized regression by Tibshirani (1996), whereby a large number of predictors are jointly modelled with a penalty term included in the likelihood to encourage sparsity. The penalty term modifies the likelihood of the regression coefficients, with a large penalty leading to the exclusion of many variables. Typically, the penalty is tuned through cross‐validation such that covariates with negligible predictive effects are removed. The over‐fitting problem is thus avoided and prediction is improved. Various extensions to the original method have been successfully applied in genomics to explore multi‐single nucleotide polymorphism (SNP) models of disease (Vignal, Bansal, & Balding, 2011; Wu, Chen, Hastie, Sobel, & Lange, 2009) or to search for master predictors (Peng, Zhu, & Bergamaschi, 2010). Bayesian versions of the LASSO have also been described (Griffin & Brown, 2010; Park & Casella, 2008) and used for efficient variable selection in genetics (Bottolo et al., 2013; Newcombe, Conti, & Richardson, 2016; Servin & Stephens, 2007; Tachmazidou, Johnson, & De Iorio, 2010; Wallace et al., 2015). Attractive features of Bayesian sparse regression include inference of posterior probabilities for each predictor, posterior inference on competing combinations, and, potentially most importantly, the possibility of incorporating prior information into the analysis. In a related approach, the over‐fitting problem has been addressed by recasting the animal model of classical quantitative genetics as a ridge regression model with a Gaussian prior on genetic effects. This does not in itself impose sparsity on the fitted model but has been extended in various ways to allow for a sparse component (Meuwissen, Hayes, & Goddard, 2001; Moser et al., 2015; Zhou, Carbonetto, & Stephens, 2013).

Although most sparse regression methods require individual level data, two frameworks have recently been proposed that allow construction of high‐dimensional polygenic risk models from meta‐GWAS summaries. “LDPred” is a sparse Bayesian regression framework in which multivariate weights are estimated using a combination of Markov Chain Monte Carlo (MCMC) and empirical Bayes (Vilhjálmsson et al., 2015). The framework is empirical Bayes in that a fixed value is estimated from the data for the residual variance (derived from a heritability estimate). A large proportion of variants are assumed to have no effect, and the proportion of causal variants is selected from a range of values according to performance in the validation data, with those variants assumed to have effects following a Gaussian distribution. The second method, “lassosum”, uses a non‐Bayesian penalized‐regression framework with a Lasso type penalty (Mak et al., 2017). The penalization parameters are optimized according to predictive performance in the validation data, similarly to LDPred, but a “pseudo‐validation” approach is also proposed to obtain near‐optimal values of the two tuning parameters. Both LDPred and lassosum account for LD using genetic correlation estimates from external reference data. In a comparative study they performed similarly, both outperformed simple approaches based on pruning and *p*‐value thresholding, which is to be expected if there are multiple causal variants in LD (Dudbridge & Newcombe, 2015).

In this work, we propose a new two‐step framework for constructing polygenic risk scores from summary data, which builds on our method “JAM”, a sparse Bayesian regression model for multivariate fine‐mapping from summary data (Newcombe et al., 2016). In the first step, models are fit independently to chromosomal blocks of limited size, and then combined in a second step to account for long‐range LD. This obviates a practical need to define LD blocks as required, for example, by LDPred, but, more pertinently, provides the flexibility to capture highly complex genetic models, since sparsity and heritability are calibrated individually within each LD block at step one. A further advantage is that our approach is extremely parallelizable, since the block‐specific analyses can be distributed across a high‐performance cluster, offering potentially much faster performance when hundreds of computing cores are available. In comparison to penalized regression approaches such as lassosum, our approach provides predictions that are model averaged, that is, they reflect uncertainty in the best selection of variants since they are averaged over competing combinations. Notably, the use of Bayesian model averaging means that all SNPs may enter the polygenic score, even though each evaluated model is sparse.

## 2 METHODS

Our aim is to build a high‐dimensional sparse regression model using summary data. We start with a brief summary of our previous “JAM” model, which allows multivariate fine‐mapping from univariate summary data, and how this framework can be used to construct polygenic risk models. Second, we describe a two‐step extension for the adjustment of long‐range LD, facilitating genome‐wide application.

### 2.1 Inference of multivariate polygenic weights from univariate summary data

We start by defining the standard multivariate linear regression of a vector of *n* trait values y on *P* variants in the columns of an *n* × *P* genotype matrix X:

(1) $$y\;\sim \;N(X\beta ,\sigma^{2})$$

β denotes the *P*‐length vector of multivariate, that is, correlation‐adjusted, genetic effects, and $\sigma^{2}$ denotes the residual variance. Note that both trait and genotypes are mean‐centred, allowing a simplification of the standard regression model to exclude the intercept term. Our previous summary data method JAM (Newcombe et al., 2016) is based on the following model:

(2) $$X\prime y\;\sim \;\mathrm{MVN}(X\prime X\beta ,X\prime X\sigma^{2})$$

which is derived from Equation (1) by multiplying through by X′. X′y, which has as many elements as genetic variants, can be derived from univariate effect estimates from regressions of each variant and the trait (Newcombe et al., 2016; Verzilli et al., 2008; Yang et al., 2012), as are reported by a typical GWAS or meta‐GWAS. Using a plug‐in estimate for the genetic correlation matrix X′X, as obtained from a reference data set, the model depicted in Equation (2) can therefore be fitted using summary data. Crucially, inference is obtained for the same correlation‐adjusted vector of genetic effects, β. To ease the computational burden, we invoke a Cholesky decomposition of X′X to map Equation (2) to a set of independent Gaussian distributions. The Cholesky decomposition of X′X provides an upper triangular and therefore invertible matrix, L, which satisfies:

$$X^{\prime }X=L\prime L$$

Multiplying Equation (2) through by $L^{\prime -1}$, we obtain:

(3) $$L^{\prime -1}X\prime y\;\sim \;\mathrm{MVN}(L\beta ,\sigma^{2})$$

that is, a model with the same form as a standard linear regression with independent residual errors.

#### 2.1.1 Sparsity inducing prior on which variants have predictive effects

To avoid over‐fitting in the context of potentially many genetic effects, we use a Bayesian sparse regression framework to draw inference under the model described by Equation (3). This is facilitated by introducing a latent binary vector $\gamma =\left(\gamma_{1},\;\ldots \;\gamma_{P}\right)$ of indices for whether each variant has a nonzero effect, that is, included in the model. Denoting the proportion of variants with nonzero effects as π, sparsity is induced via a β prior distribution:

(4)  π ~ Beta(1, λP)

This prior formulation is widely used in Bayesian variable selection due to the intrinsic correction for multiplicity; the marginal prior odds of any single variable having an effect is 1/λP, and therefore decreases with the total number of variables *P*, whereas the global prior odds of any effect is constant at 1/λ (Wilson, Iversen, Clyde, Schmidler, & Schildkraut, 2010). λ can be chosen to induce more or less sparsity, depending on prior beliefs. In practice, we recommend trying a range of several λ and picking the value which optimizes predictive performance in the validation data. For the analyses presented in the results, we tried λ=0.001,⁢  0.01,⁢  0.1,⁢  1. Note that λ is a hyper‐parameter for the distribution of π, the proportion of variants with nonzero effects, and conditional on λ, we allow the proportion π to be random; thus we allow greater flexibility than approaches that consider a set of fixed values for π (Vilhjálmsson et al., 2015)

#### 2.1.2 Prior on variant effects

Conditional on a selection of variants indicated in γ, we place a hierarchical normal prior over the corresponding subvector of multivariate effects, which we denote by $\beta_{\gamma }$:

$$\beta_{\gamma }\;\sim \;N(0,\;{I\sigma }_{\beta }^{2})$$

$\sigma_{\beta }^{2}$ may be interpreted as the variance among the “true” genetic effects. To estimate this crucial parameter largely from the data, we assign a vague hyper‐prior (rather than choosing a fixed value), which we have used previously in an ′omics setting (Newcombe et al., 2014):

$$\sigma_{\beta }\;\sim \;\mathrm{Unif}\left(0.05,2\right)$$

Our polygenic prediction model is completed with a prior on the residual variance, $\sigma^{2}$. We use a standard vague prior:

$$\sigma^{2}\;\sim \;\mathrm{Inv}-\mathrm{Gamma}\left(0.01,0.01\right)$$

For many traits, there will be prior information available on the heritability, and therefore on the residual variance of a whole genome predictor. However, as we explain below, in the first step of our parallelized approach, the algorithm is only applied to a small number of SNPs simultaneously. Hence the use of the vague priors above.

#### 2.1.3 Binary traits

So far, our framework has assumed the trait of interest is continuous. This is because we rely on a linear modelling framework to relate univariate summaries to multivariate effects via the linear transformation X′y. In the case of binary traits, we derive X′y after first mapping univariate log‐odds ratios to approximate linear effects via their *z*‐scores. That is, we infer the univariate effects that would have been estimated if the binary outcome has been modelled by linear regression, allowing construction of X′y. This strategy is employed in other linear‐based summary data frameworks (Chen et al., 2015) including LDPred (Vilhjálmsson et al., 2015), to which we refer readers for a detailed description of this mapping.

#### 2.1.4 Inference of a posterior model averaged polygenic risk score via reversible jump Markov Chain Monte Carlo

We cannot derive analytical expressions for the posterior of β and so use reversible jump MCMC (Green, 1995) to sample from the required posterior distribution. The reversible jump sampling scheme starts with an initial model, γ(0), which is a selection of variants in Step 1 and corresponding parameter values, θ(0). To sample the next model and set of parameters, which we denote by γ(1) and θ(1), we propose moving from the current state to another model and/or parameter values, γ* and $\theta^{*}$, using a proposal function q($\gamma^{*},\theta^{*}\;|\;\gamma ,\theta )$. The proposed model and parameters are accepted with probability equal to the Metropolis‐Hastings ratio:

$$\mathrm{MHR}=\frac{P\left(D|\;\gamma^{*},\theta^{*}\right)P\left(\theta^{*}|\;{\;\gamma }^{*}\right)P(\gamma^{*})}{P\left(D|\;\gamma ,\theta \right)P\left(\theta \,\;|\;\gamma \right)P(\gamma )}\times \frac{q(\gamma ,\theta \;|\;\gamma^{*},\theta^{*})}{q({\gamma^{*},\theta }^{*}|\;\gamma ,\theta )}$$

where ***D*** is the observed data, and P(D|γ,θ) is the multivariate likelihood described by Equation (3). $P(\gamma^{⁎})$ is the β‐binomial model space prior defined in Equation (4) and $P\left(\theta^{*}|\gamma^{*}\right)$ is the prior on the parameters conditional on (i.e. included in) the model. The proposed model and parameter values are therefore accepted with a probability proportional to both their likelihood and prior support. If this new set of values is accepted, we set γ(1) = γ* and $\theta \;\left(1\right)=\theta^{*}$, otherwise they are discarded and the current values are retained; γ(1) = γ(0) and θ(1) = θ(0). It can be shown that this produces a sequence of parameter/model samples, which converge to the target posterior distribution (Green, 1995). After obtaining a posterior sample of effects, β, for each variant (note that many of these values may be zero corresponding to exclusion from the model at a particular iteration), we average to obtain the final weighted polygenic risk score across all variants, $\hat{\beta }$. This may also be interpreted as the vector of posterior mean variant effects, averaged over the posterior distribution of models.

#### 2.1.5 Two‐step approach to the adjustment of long‐range linkage disequilibrium

A practical limitation to the use of the method outlined above is that a full rank genotype matrix is required to construct the plug‐in estimate for X′X. This is due to the necessary inversion during inference (Newcombe et al., 2016). Therefore, the number of variants that can be simultaneously modelled must necessarily be less than the number of individuals in the reference data, and, in practice, depending on the amount of correlation, considerably less. In fine‐mapping applications, this is generally not a problem. In the genome‐wide context, Mak et al. (2017) regularized X′X with a further penalty parameter, transforming their original Lasso model to an elastic net problem. LDpred uses a Gibbs sampler that essentially models a sliding window of variants, of fixed size. Here we suggest the following two‐step approach to build models within blocks, under the Bayesian sparse regression outlined above, and then account for cross‐block correlation to arrive at a genome‐wide model.

##### Step 1: Block‐specific polygenic scores

In step one, the *P* variants genome‐wide are split into *B* small blocks of 100 variants each, and JAM is used to derive posterior mean weights for the variants within each block: ${\hat{\beta }}_{b}$ for b=1,.. B. For the analysis of each block *b*, the input data is the set of marginal variant effects as well as the columns of the reference genotype matrix corresponding to the variants in block *b*. The block size choice of 100 could be varied but we found that 100 worked well in practice (see Section 3).

##### Step 2: Between‐block adjustment

In the absence of correlations across blocks, an unbiased genome‐wide polygenic risk score, with weights which we denote by ${\hat{\beta }}_{G}$, could then be constructed by simply appending the block‐specific scores:

$${\hat{\beta }}_{G}=({\hat{\beta }}_{1},\;..,{\hat{\beta }}_{B})$$

However, this risk score will be biased in the presence of correlations *across* blocks, since they were ignored in Step 1. To account for *cross‐block* correlations, we introduce a second layer of multivariate weights, $\delta \;=\;(\delta_{1},\;\;.\;.\;\delta_{B})$, which adjust the “marginal” block‐specific scores for one another. Specifically, we seek to estimate the “block‐adjusted” risk score:

$${\hat{\beta }}_{G}=(\delta_{1}\;{\hat{\beta }}_{1},\;..\;\delta_{B}\;{\hat{\beta }}_{B})$$

It is instructive to consider the block‐specific scores as a set of *B* covariates, and view δ as the multivariate vector of effects, we would obtain from a regression of y on an *n* × *B* matrix of the block‐specific scores, S. For clarity, the element of S corresponding to individual *i* and block *b* is:

(5) $$s_{i,b}=\sum_{p=1}^{100}x_{i,b,p}\;{\hat{\beta }}_{b,p}$$

where $x_{i,b,p}$ is their genotype at variant p in block *b*, and ${\hat{\beta }}_{b,p}$ is the corresponding weight from the block‐specific score as estimated in Step 1. It transpires that the estimation of δ is straightforward, by reapplying the same methodology used to estimate multivariate SNP weights for each block in Step 1, except now we wish to adjust the “marginal” block‐scores for the block‐block correlation structure S′S. The analogy of Equation (2) is:

(6) $$S\prime y\;\sim \;\mathrm{MVN}(S^{\prime }S\delta ,S\prime S\sigma^{2})$$

By applying Equation (5) to the reference matrix X, we also obtain a plug‐in estimate for S′S. In the same way X′y for Equation (2) is constructed from the marginal variant effects (and their minor allele frequencies) we can construct S′y from the marginal block‐score “effects” and the column means of S. By construction, the “marginal” effect of each block‐specific score is 1; a unit increase in each risk score is associated with the same unit increase in the trait y. Intuitively, all scores have equivalent unit effects on y because the variant‐specific effects from which they are composed are on the same scale as per Equation (1), which defines the unit SNP effects within each block. If a block contains variants with small effects, a large number is required for a score of 1. Conversely, for a block containing larger effects, fewer variants are required to achieve the same score. We obtain a maximum likelihood estimate for $\hat{\delta }$ after multiplying both sides of Equation (6) through by the inverse Cholesky decompositon of correlation matrix S′S to obtain a linear regression analogous to Equation (3). Note that model selection is not carried out in Step 2 since sparsity among the genetic effects has already been imposed in Step 1 under the prior in Equation (4), and we do not expect sparsity at the block level. Obtaining $\hat{\delta }$ completes our genome‐wide polygenic score in which correlations are accounted for both within and across regions:

$${\hat{\beta }}_{G}=({\hat{\delta }}_{1}\;{\hat{\beta }}_{1},\;..\;{\hat{\delta }}_{B}\;{\hat{\beta }}_{B})$$

Note that the first step of our algorithm is why it is highly parallelizable. Although, in principle, joint analysis of multiple blocks would help inform estimation of the residual variance, $\sigma^{2}$, as well as the proportion of “causal” variants, θ, in practice we found no difference compared to estimating each block‐specific score, ${\hat{\beta }}_{b}$, independently. By running the block‐specific analyses independently, we could take advantage of large numbers of CPU cores available to us via a high performance computing cluster (HPC). In each of the following real data applications we ran our algorithm for 200,000 iterations in the Step 1 block‐specific analyses.

## 3 RESULTS

### 3.1 Cross‐validation in the Welcome Trust Case Control Consortium

We first compared performance of our proposed method against LassoSum and LDPred using individual level genotypes, measured using the 500K Affymetrix Chip, for seven traits in the Welcome Trust Case Control Consortium (WTCCC, 2007). In total, data were available for 2,835 common controls, 1,827 bipolar disorder, 1,880 coronary artery disease (CAD), 1,684 Crohn's disease, 1,904 hypertension, 1,834 rheumatoid arthritis, 1,933 type 1 diabetes (T1D), and 1,872 type 2 diabetes cases, respectively. For each trait, the WTCCC cases and controls were randomly partitioned such that 2/3rd of the samples were used to train all models and 1/3rd of the samples were used for testing. The random partitioning was conducted in a stratified manner, such that each of the three folds had the same proportion of cases and controls. After pruning variants with missing rates above 1%, and for maximum LD below r^2^ 95% using the Plink software package (Purcell et al., 2007), we were left with between 255,781 and 256,925 variants for each trait. LDPred and lassosum were run with default parameters as described in their papers. Results are presented in Table 1. Out‐of‐sample predictive results were pooled across all three folds before calculating the final performance summaries. 95% confidence intervals (95% CI) for the receiver operating characteristic area under the curves (ROC AUCs) were calculated using 2,000 stratified bootstrap replicates and the pROC R package. We also checked results using a different cross‐validation partitioning seed, which were indistinguishable (not shown).

**Table 1:** Application of three summary statistics prediction methods in the Welcome Trust Case Control Consortium under three‐fold cross‐validation

Trait	LassoSum		LDPred		JAM	
	AUC	r^2^	AUC	r^2^	AUC	r^2^
Bipolar disorder	0.67 (0.64, 0.69)	0.09	0.66 (0.63, 0.69)	0.08	0.70 (0.67, 0.73)	0.13
Coronary artery disease	0.59 (0.56, 0.62)	0.02	0.59 (0.56, 0.62)	0.02	0.65 (0.62, 0.67)	0.08
Crohn's disease	0.65 (0.62, 0.68)	0.07	0.69 (0.66, 0.72)	0.10	0.69 (0.66, 0.72)	0.12
Hypertension	0.61 (0.58, 0.64)	0.04	0.59 (0.56, 0.62)	0.03	0.58 (0.55, 0.61)	0.02
Rheumatoid arthritis	0.71 (0.68, 0.73)	0.12	0.72 (0.69, 0.75)	0.14	0.74 (0.71, 0.76)	0.16
Type 1 diabetes	0.83 (0.80, 0.85)	0.30	0.87 (0.85, 0.89)	0.39	0.86 (0.84, 0.88)	0.36
Type 2 diabetes	0.62 (0.60, 0.65)	0.04	0.60 (0.57, 0.63)	0.05	0.64 (0.61, 0.67)	0.08

*Note*: ROC AUCs and predictive r^2^ are presented, with ROC AUC 95% confidence intervals calculated via 2,000 stratified bootstrap samples. For each method, performance is presented for the best performing sparsity.

Abbreviations: AUC, area under the curve; ROC, receiver operating characteristic.

For most traits, the performance of LDPred and LassoSum was similar, although LDPred offered improvements for Crohn's disease, AUC of 0.69 (95% CI: 0.66–0.72) versus 0.65 (95% CI: 0.62–0.68), and T1D, AUC of 0.87 (95% CI: 0.85–0.89) versus 0.83 (0.80–0.85). Our two‐step JAM method appeared the most robust, generally resulting in performance on par with the best performing of LDPred and lassosum. Runtimes for our two‐step approach ranged between 3 and 4 min when running different prior settings and chromosomes in parallel on different computing nodes, with block parallelization across the 16 cores of each compute node. These runtimes were similar, though slightly faster than lassosum, which typically took an extra minute to run. Conversely, LDPred took several hours. Therefore, with a large numbers of compute cores available, our parallelizable approach was the fastest, while offering typically better predictive performance. However, in terms of total computational cost, LassoSum is the most efficient method, achieving these runtimes when only a single compute node is available.

### 3.2 Meta‐GWAS applications for type 1 diabetes, coronary artery disease, and schizophrenia

Next, we exemplify our method in three case studies using summary statistics from meta‐GWAS for T1D (T1DGC; 3,983 cases, 3,999 controls), CAD (cardiogram; 40,170 cases, 97,365 controls), and schizophrenia (PGC; 34,241 cases, 45,604 controls). Summary statistics from the T1DGC study were available for both genotyped SNPs (Illumina 550K) as well as imputed SNPs unique to the older Affymetrix 500K array. The imputation, originally conducted to facilitate meta‐analysis with the WTCCC, was based on a substantial subset of controls for which both arrays were available. Details of the original QC, imputation method, and meta‐analysis can be found in Barrett et al. (2010). The cardiogram summary statistics came from a meta‐analysis after excluding the WTCCC and restricting to the 37 remaining studies with white European ancestry. Studies contributed either genotypes from the Metabochip array or GWAS data imputed using HapMap; further details on QC, imputation, and the meta‐analysis may be found in Nikpay et al. (2015). The schizophrenia summary statistics correspond to a meta‐analysis of 46 European studies from the Psychiatric Genomics Consortium (PGC), each of which contributed both genotyped SNPs (from various arrays) as well as imputed SNPs using a 1,000 genome reference panel. Further details of the QC, imputation, and meta‐analysis may be found in Ripke et al. (2014). Case and control samples from the WTCCC were used as independent testing data for CAD (*n* = 4,715) and T1D (*n* = 3,308). For the latter, the 1958 birth cohort was removed since these samples were used as controls in the T1DGC. For schizophrenia, testing data comprised genotypes measured using the Affymetrix 6.0 array in 5,334 samples from the Molecular Genetics of Schizophrenia (MGS) study (Shi et al., 2009). Since the MGS results were included in the PGC meta‐GWAS, we excluded their influence using a technique detailed by Mak et al. (2017), whereby the hypothetical meta‐analysis of the PGC excluding MGS is inferred according to the results from each. In each case study, the SNPs used for polygenic model building were the subset that appeared in the intersection of SNPs available in both the summary statistics and testing datasets, and remained after pruning for less than 1% missingness and LD less than r^2^ 95% in the corresponding testing data. This left 231,510 SNPs for T1D, 211,263 for CAD, and 385,474 for schizophrenia.

All three sparse regression methods—JAM, LDPred, and lassosum—were run with the same parameters as described in the WTCCC cross‐validation analyses above. We also compared against a simple *p*‐value thresholding approach, as well as a combined clumping and *p*‐value thresholding approach, which selectively removes less significant SNPs to reduce LD (Wray et al., 2014). For the *p*‐value thresholding, we used the set of *p*‐values {5e‐8, 1e‐5, 1e‐4, 1e‐3, 0.0015, 0.0025,…0.995}, and when combined with clumping, we used r^2^ thresholds of 0.2, 0.5, and 0.8.

**Figure 1:**
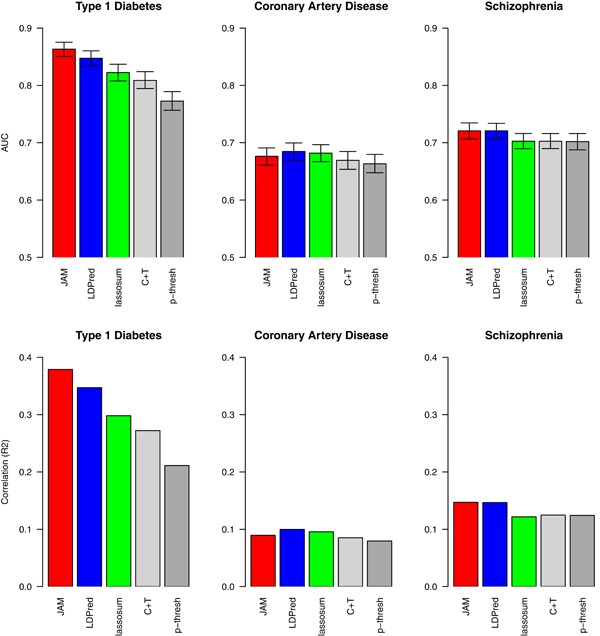
Receiver operating characteristic area under the curves ROC AUCs and predictive r^2^ for various predictive methods in three case studies training polygenic predictive models using meta‐GWAS summaries. For type 1 diabetes, the T1DGC (*n* = 8,005) was used for training and the Welcome Trust Case Control Consortium for validation. For coronary artery disease (CAD), cardiogram (*n* = 137,535) was used for training and the WTCCC for testing. For schizophrenia, the Psychiatric Genomics Consortium (*n* = 74,511) was used for training and the MGS study for testing. For each analysis and method, results are presented for the best performing sparsity. ROC AUC 95% confidence intervals were calculated using 2,000 stratified bootstrap replicates

For T1D, all sparse regression methods surpassed simpler *p*‐value thresholding, with JAM offering the best predictive performance; r^2^ of 0.38 compared to 0.35 for LDPred and 0.30 for lassosum, AUC of 0.86 (95% CI: 0.85–0.88) compared to 0.85 (95% CI: 0.83–0.86) for LDPred, and 0.82 (95% CI: 0.81–0.84) for Lassosum. We suspect the improved performance from JAM is due to the large number of correlated signals in regions of both short‐ and long‐range LD within the major histocompatibility complex (MHC) on chromosome 6, for which a more sophisticated model search and averaging algorithm should offer greater accuracy. To confirm, we reran JAM, LDPred, and lassosum after excluding chromosome 6 from the training data. As expected, performance was considerably diminished for all three methods but was indistinguishable between JAM, LDPred, and lassosum (r^2^ of 0.08 and AUC of 0.66 for all), indicating that JAM's improved performance for T1D is indeed driven by a more flexible model for the MHC. For schizophrenia and CAD, where the polygenic signal is weaker and more dispersed through the genome, all methods performed similarly (Figure 1).

**Figure 2:**
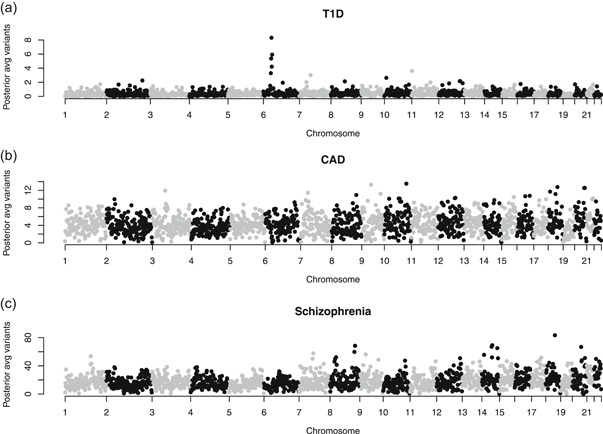
Block‐specific posterior mean numbers of selected single nucleotide polymorphisms by the JAM method in each of the three meta‐GWAS case studies. The vertical spread indicates the variation in block‐specific adapted sparsities from step one of our proposed framework. Note that the global average varies across the case studies owing to different optimal λs, which control the global sparsity. For CAD and schizophrenia, summary statistics were available from considerably larger training datasets, allowing the estimation of many more small effects (see Table 2). CAD, coronary artery disease

A unique feature of our method is the ability to adapt sparsity within local SNP blocks. To demonstrate how this looked in practice, we plotted the posterior mean selected number of variants for each block from each case study (Figure 2). As expected for T1D, a number of blocks within the MHC on chromosome 6 had considerably more SNPs selected, indicating that JAM adapted to impose less sparsity, in a region containing strong signals. For CAD and schizophrenia, the spread of block‐specific selections was more similar across chromosomes, but there was still variation, demonstrating block‐to‐block adaption. Note that the larger number of total SNPs selected for CAD and schizophrenia was due to much larger training samples, which allowed the estimation of many more smaller effects—see Table 2. The pattern of runtimes across all three case studies was similar to the WTCCC cross‐validation study above, with JAM offering the best runtimes when different priors and chromosomes were run in parallel, lassosum not far behind, and LDPred significantly slower (Table 2).

**Table 2:** Computational aspects of JAM and runtimes (in minutes) of the different methods applied to the meta‐GWAS case studies

Case study	Total	JAM	JAM	JAM	lassosum	LDPred
	SNPs	λ	SNPs	Runtime	Runtime	Runtime
T1D	231,510	0.01	712	4.6	10.3	61.5
CAD	211,263	0.001	7233	5.1	7.5	27.5
Schizophrenia	385,474	1E‐04	30544	16.6	17.4	157.2

*Note*: The total number of SNPs analyzed (i.e., after QC), for all methods, is shown in the first column. The next two columns correspond to the optimal value of for use with JAM—smaller values encourage more sparsity—and the posterior average number of SNPs selected into the corresponding optimal JAM model

Abbreviations: CAD, coronary artery disease; SNP, single‐nucleotide polymorphism; T1D, type 1 diabetes.

## 4 DISCUSSION

We present a novel, flexible, and parallelizable approach to the construction of genome‐wide polygenic risk scores from meta‐GWAS summary statistics. In cross‐validation analyses of all seven traits in the WTCCC, and three case studies using meta‐GWAS summary results, our method offered similar predictive performance to two alternative approaches, lassosum and LDPred, while achieving the fastest runtimes. The improved runtimes were due to the highly parallelizable nature of our algorithm, whereby genetic correlations are adjusted for in two steps, allowing the analysis of many small groups of SNPs independently at Step 1, in which we only account for short‐range LD. Because we partition the genome into blocks rather arbitrarily at Step 1, we allow for LD straddling block boundaries, as well as longer range LD, in Step 2. The adjustment for both short‐ and long‐range LD is novel to our method, and is more satisfactory than simple block‐wise model fitting since LD decays stochastically and any attempt to partition the genome will result in some correlations across blocks. The most important instance of long‐range LD in the genome is in the human leukocyte antigen (HLA) complex, and it is those diseases with HLA associations where our method, and others that account for LD, did best. There is a particularly strong and complex HLA signal for T1D, consisting of multiple heterogeneous signals in regions of both short‐ and long‐range LD, and it was here, in both the WTCCC and T1DGC case studies, that performance gains were greatest compared to LDPred and lassosum, which only model short‐range LD. A further novel feature of our method, compared to LDPred and lassosum, is that we allow for different genetic models, that is, signal sparsities, across different blocks. This is achieved by treating sparsity and local heritability as random quantities rather than fixed hyperparameters, which should offer increased statistical robustness over repeated replication studies, as well as inference on where highly polygenic predictive signals are concentrated (see Figure 2).

Although we demonstrate the two‐step approach in a Bayesian model averaging framework, the idea is generic to regression and could be leveraged for gains in other frameworks too. There are, however, some conceptual advantages to using a formal Bayesian model averaging approach. First, we do not have to fix an assumed proportion of causal variants, but instead treat this as an unknown parameter with a prior that is integrated over separately for each block. Consequently, we observed that predictive performance under different priors was more robust than, analogously, LDPred and lassosum across the range of their respective sparsity tuning parameters. Although the standard practice is to choose the sparsity tuning parameter for each method according to best performance in the test data, as we do here, this will, in principle, lead to a degree of optimism, that is, overfitting. Ideally, an independent data set should be used to select tuning parameters such that the predictive algorithm is entirely finalized before application in the test data. This was not practical in the data sets we considered here, but the observation that our fully Bayesian approach is more robust to the sparsity choice provides confidence that results from the case studies are less likely to suffer from optimism. A further appeal of our approach is that it provides a unified analytical model for fine mapping and polygenic score estimation. In the fine mapping context, the genetic effects can be integrated out as nuisance parameters (Newcombe et al., 2016), with the objects of inferences being the probabilities of including each SNP in the model. Here, by contrast, but under the same framework, the SNP effects are obtained by averaging over the inclusion probabilities.

Further flexibility is possible through the prior on effect sizes for selected SNPs. We have used a conjugate Gaussian prior, which after averaging over sparse models leads to a marginal posterior distribution similar to that obtained from a slab‐and‐spike prior. Thus our approach is conceptually similar to LDpred at the block level, but we allow for different posterior distributions across blocks, related by a common vague prior distribution. Now our approach can readily be extended by letting each SNP belong to one of several classes, with class membership probabilities drawn from a multinomial distribution and distinct Gaussian distributions for the effects in each class. After model averaging, this would be analogous to some mixture models recently proposed for individual level data (Moser et al., 2015; Zhou & Stephens, 2014), with the advantage that we can use summary statistics and fit distinct models to blocks of SNPs in parallel. Thus, while the present work provides a proof of principle with comparable accuracy to competing methods for summary statistics, it is readily extensible in ways that mirror the best current models for individual level data. Furthermore, a mixture modelling formulation may provide a natural way to accommodate external SNP‐specific functional annotation information from public resources, such as annotation databases, expression, and methylation quantitative trait locus analyses. An extension is conceivable whereby SNP‐specific functional annotations would influence class assignments within the mixture of Gaussian distributions. For example, rather than using a naïve multinomial distribution, an informative β prior could be constructed across the class assignment ratios. Indeed, it has previously been shown that reflection of prior annotation when constructing polygenic risk scores is advantageous (Shi et al., 2016). We plan to pursue all these ideas in future work.

Assuming availability of a large number of computing nodes, our method offered the fastest performance with similar predictive performance to lassosum and LDPred. The level of computing resources required for these runtimes (~100 compute nodes) is available to many researchers today, however, our parallelization approach can take advantage of even more resources as they inevitably become available over the coming years, since many of the individual block analyses are still being run sequentially in Step 1. With sufficient computing resources, in principle, every single 100 SNP block could be run be run in parallel, which would reduce runtimes to well under a minute in our three case studies. Relatedly, while we found the use of 100 SNP blocks in Step 1 worked well in practice, this choice is likely to be dependent on marker density, which will determine the average genomic length of these blocks. Using microarray data, as in our case studies, the 100 SNP blocks span genomic lengths the order of 100 kb for microarray data, but, when, for example, using imputed sequence data larger blocks may lead to better performance. We intend to explore this in more detail in future work.

We have incorporated our algorithm “JAMPred” into our existing fully documented R package for Bayesian model selection “R2BGLiMS”, which also contains the original “JAM” software, and is freely available to download via github https://github.com/pjnewcombe/R2BGLiMS. We have included scripts demonstrating the JAMPred syntax, and how to distribute a genome‐wide analysis on an HPC, in the Supporting Information.

## Supporting information

Supporting information

## Data Availability

Data sharing is not applicable to this article as no new data were created or analyzed in this study.
