# Supplementary material for: A flexible and parallelizable approach to genome‐wide polygenic risk scores
Source: Genet Epidemiol. 2019 Jul 22;43(7):730–41. doi: 10.1002/gepi.22245 (PMC6764842; doi:10.1002/gepi.22245)
Supplement: Supplementary file 1 — Supporting information [file GEPI-43-730-s001.pdf]

Supplementary Material for:

"A flexible and parallelisable approach to genome-wide polygenic risk scores"

Paul J Newcombe[1*], Christopher P Nelson[2,3], Nilesh J Samani[2,3], CARDIoGRAMPlusC4D, and Frank Dudbridge[4]

Affiliations:
[1]MRC Biostatistics Unit, School of Clinical Medicine, Cambridge Institute of Public Health, Forvie Site, Robinson Way, Cambridge Biomedical Campus, Cambridge, CB2 0SR, UK
[2]Department of Cardiovascular Sciences, University of Leicester, Cardiovascular Research Centre, Glenfield Hospital, Leicester, LE3 9QP, UK
[3]NIHR Leicester Biomedical Research Centre, Glenfield Hospital, Groby Road, Leicester, LE3 9QP, UK
[4]Department of Health Sciences, Centre for Medicine, University of Leicester, Leicester LE1 7RH, UK
*Corresponding author: paul.newcombe@mrc-bsu.cam.ac.uk

Running JAMPred within the R2BGLiMS package

To install R2BGLiMS, which facilitates Bayesian variable selection and model averaging for a variety of models, you need to have a Java JDK installed on your system. Note that a Java runtime environment alone is not sufficient. The Java JDK may be downloaded from: https://www.java.com/download/. R2BGLiMS may be installed from github as follows (requires the "devtools" library):

```
install.packages("devtools")
library(devtools)
install_github("pjnewcombe/R2BGLiMS")
```

The following examples may also be found by typing `?JAMPred` after loading the R2BGLiMS package:

```
# --- Load libraries
library(R2BGLiMS)
data("JAMPred_Example")

#################################################################
# --- 1) Simple demonstration of syntax for binary traits --- #
#################################################################

# Run JAMPred
snps <- chromosome.snps[[1]] # Only use chromosome 1 data
jampred.res.bin <- JAMPred(
  marginal.betas = marginal.logors[snps],
  n.training = n.training,
  marginal.logor.ses = marginal.logor.ses, # Only necessary for a binary trait
  p.cases.training = n.cases.training/n.training, # Only necessary for a binary trait
  ref.geno = data.validation[,snps],
  total.snps.genome.wide = 500000, # Total SNPs across all chromosomes
  n.mil = 0.2,
  seed = 1 # For re-producibility. If not set a random seed is used
)

# Generate predictions
out.of.sample.predictions <-
  data.validation[,jampred.res.bin$snps] %*%
  jampred.res.bin$step2.posterior.mean.snp.weights

# Predictive r2
cor(out.of.sample.predictions, data.validation[,"d"])^2 # Should be 0.18

#################################################################
# --- 2) Simple demonstration of syntax for binary traits --- #
#################################################################

# Run JAMPred
snps <- chromosome.snps[[1]] # Only use chromosome 1 data
jampred.res.lin <- JAMPred(
  marginal.betas = marginal.ctsbetas[snps],
  n.training = n.training,
  ref.geno = data.validation.cts[,snps],
  total.snps.genome.wide = 500000, # Total SNPs across all chromosomes
  n.mil = 0.2,
  seed = 1 # For re-producibility. If not set a random seed is used
)

# Generate predictions
out.of.sample.predictions <-
  0 + # NB: A trait mean could be set here (otherwise it is assumed the outcome is mean-centred)
  data.validation.cts[,jampred.res.lin$snps] %*%
  jampred.res.lin$step2.posterior.mean.snp.weights

# Predictive r2
cor(out.of.sample.predictions, data.validation.cts[,"y"])^2 # Should be 0.15
```

Running JAMPred across different chromosomes and sparsity settings

We now demonstrate a simple analysis using JAMPred to model association data from two different chromosomes, and using different choices of the sparsity parameter λ. The synthetic data included in `JAMPRED_Example` contain genetic associations for 800 SNPs, split across 2 chromosomes with 400 SNPs each.

In this simple demonstration of the syntax, we model each chromosome/sparsity combination one after another in a loop. In practice we would recommend parallelising these analyses over an HPC (see the next section). Data for a complete chromosome should be passed to each individual JAMPred call, in order to fully account for LD in JAMPred's step 2 adjustment. Each JAMPred call automatically parallelises modelling the SNP blocks within the over the specified number of available CPU cores (the default is 2). In the example below, JAMPred splits the SNPs on each chromosome into 4 x 100 SNP blocks (the default block size) and utilises two CPU cores to analyse the blocks in parallel pairs. **NB:** JAMPred does not require a separate CPU core for every SNP block in the chromosome – it is designed to jointly model a set of blocks on each core – the user simply needs to specify the number of CPU cores available to each call of JAMPred (on an HPC this might be 28 or more) and let JAMPred divide the blocks accordingly.

```
#####################################################################################
# --- 3) Using JAMPred to model multiple chromosomes under different sparsities --- #
#####################################################################################

# Run JAMPred, looping over sparsities and chromosomes
jampred.res.loop <- list() # List to hold all the results
for (lambda in c(0.01, 0.1, 1, 10)) { # Loop over lambda choices (see the paper)
  jampred.res.loop[[paste(lambda)]] <- list()
  for (chr in c(1:2)) { # Loop over chromosomes
    jampred.res.loop[[paste(lambda)]][[chr]] <- JAMPred(
      marginal.betas = marginal.logors[chromosome.snps[[chr]]],
      n.training = n.training,
      marginal.logor.ses = marginal.logor.ses,
      p.cases.training = n.cases.training/n.training,
      ref.geno = data.validation[,chromosome.snps[[chr]]],
      total.snps.genome.wide = 500000,
      n.mil = 0.2,
      beta.binom.b.lambda = lambda,
      initial.block.size = 100, # Default size of SNP blocks
      seed = 1 # For re-producibility. If not set a random seed is used
    )
  }
}

# Generate predictions for each lambda, summing predictive scores over chromosoms
out.of.sample.predictions <- list()
for (lambda in c(0.01, 0.1, 1, 10)) {
  out.of.sample.predictions[[paste(lambda)]] <- 0
  for (chr in c(1:2)) {
    out.of.sample.predictions[[paste(lambda)]] <-
      out.of.sample.predictions[[paste(lambda)]] +
      data.validation[,jampred.res.loop[[paste(lambda)]][[chr]]$snps] %*%
      jampred.res.loop[[paste(lambda)]][[chr]]$step2.posterior.mean.snp.weights
  }
}

# Predictive r2
sapply(out.of.sample.predictions, function(preds) cor(preds,data.validation[,"d"])^2)
# Predictive correlation 0.37 and highest at lambda = 0.01
```

Running JAMPred on a High Performance Computer Cluster using the SLURM job submission system

Here we demonstrate the use of JAMPred to efficiently model genome-wide association data via parallelisation over a High Performance Computer (HPC) cluster. You will need R2BGLiMS installed and run-able from your HPC user account.

The overall JAMPred analysis is split into a series of `jobs', which are then distributed across the `nodes' and CPUs available on the HPC. In this example, and how we typically run JAMPred, each `job' corresponds to the modelling of data corresponding to a specific chromosome and with a specific choice of the sparsity parameter λ. JAMPred further parallelises each of these analyses over LD blocks, using the CPU cores available on the node. Four scripts are used:

1) `JAMPred.R` – The R script used to run the specific chromosome/sparsity job
2) `RunJAMPred.Sh` – A shell script to submit a single job to the SLURM system
3) `BatchRunJAMPred.sh` – A shell script to submit the complete batch of jobs
4) `ProcessJAMPredBatchResults.R` – A simple script to gather the results

1) JAMPred.R

Runs a JAMPred analysis for a specific chromosome/ λ combination, indicated via the command line arguments (`commandArgs`) passed from `BatchRunJAMPred.sh` -> `RunJAMPred.Sh` -> `JAMPred.R`.

```
# Extract command line arguments
args <- unlist(strsplit(commandArgs(trailingOnly=T)[1],split="_"))
lambda <- 100*10^(-as.numeric(args[1]))
chr <- as.numeric(args[2])

# Load data
load(paste0("DataPath/data_common.RData")) # Common data e.g. P, n, validation data etc
load(paste0("DataPath/data_chromosome",chr,".RData")) # Load chromosome specific data

# Run JAMPred
jam.pred.res <- JAMPred(
  marginal.betas = marginal.logors[chromosome.snps[[chromosome]]],
  marginal.logor.ses = marginal.logor.ses,
  n.training = n.training,
  p.cases.training = p.cases.training,
  ref.geno = data.validation[,chromosome.snps[[chromosome]]],
  total.snps.genome.wide = P,
  n.mil = 0.2,
  beta.binom.b.lambda = lambda)

# Save results
save(jam.pred.res,
     paste0("ResultsPath/JAMPred_lambda",beta.binom.b.lambda,"chromosome",chr,".RData"))
```

2) RunJAMPred.sh

Runs a single instance of JAMPred.R, as a SLURM job. SLURM options are set at the top of the script. Your HPC may require additional options (e.g. the partition to run on and account to charge to).

```
#!/bin/bash
#SBATCH --nodes=1
#SBATCH --time=2:00:00 # Good practice to set an upper limit
cd YourDirectory
R --vanilla --slave --args ${R_ARGS} < JAMPred.R # R_ARGS comes from BatchRunJAMPred.sh
```

3) BatchRunJAMPred.sh

Submits a batch of jobs to the HPC, one for every sparsity setting and chromosome number.

```
#!/bin/bash
cd YourDirectory
for lambda in 1 2 3 4; do # Four different sparsity settings
  for chr in `seq 1 22`; do
      R_ARGS="${lambda}_${chr}"
      NAME=JAMPred_${R_ARGS}
      sbatch \
      -o ./${NAME}.o \
      -e ./${NAME}.e \
      -J ${NAME} \
      --export R_ARGS=${R_ARGS} \ # R_ARGS is passed through to RunJAMPred.sh
      ./RunJAMPred.sh
  done
done
```

4) ProcessJAMPredBatchResults.R

Simple script to merge results from the different batch analyses

```
load(paste0("DataPath/data_common.RData")) # Common data e.g. P, n, validation data etc


# Generate predictive scores for every lambda, summing over chromosome specific scores
out.of.sample.predictions <- list() # List of out of sample scores for each lambda
for (lambda in c(0.01, 0.1, 1, 10)) {
  out.of.sample.predictions[[paste(lambda)]] <- 0
  for (chromosome in c(1:22)) {
    load(paste0("ResultsPath/JAMPred_lambda",lambda,"chromosome",chr,".RData"))
    out.of.sample.predictions[[paste(lambda)]] <-
      out.of.sample.predictions[[paste(lambda)]] +
      data.validation[,jam.pred.res$snps] %*%
      t(t(jam.pred.res$step2.posterior.mean.snp.weights))
  }
}

# Out of sample predictive correlation for every lambda
sapply(out.of.sample.predictions, function(preds) cor(preds,data.validation[,"d"])^2)
```
